# Functional and Structural Differences of Brain in Patients With Vestibular Migraine: A Resting‐State Functional MRI and DTI Study

**DOI:** 10.1002/brb3.70569

**Published:** 2025-06-12

**Authors:** Ni Liu, Qijun Yu, Shaowei Gan, Yonghui Pan, Zhaowen Qiu

**Affiliations:** ^1^ Department of Neurology The First Affiliated Hospital of Harbin Medical University Harbin China; ^2^ College of Computer and Control Engineering Northeast Forestry University Harbin China; ^3^ Key Laboratory of Hepatosplenic Surgery, Ministry of Education, The First Affiliated Hospital of Harbin Medical University, Harbin China; ^4^ Heilongjiang TuoMeng Technology Co., Harbin China

**Keywords:** DTI, fMRI, vestibular migraine

## Abstract

**Introduction:**

The pathogenesis of vestibular migraine (VM) still remained unclear. This study used functional magnetic resonance imaging (fMRI) and diffusion tensor imaging (DTI) techniques to study the characteristics of VM brain structure and function changes, to explore the association between those changes and clinical symptoms.

**Methods:**

13 VM patients and 12 migraineurs were recruited from a tertiary hospital, while 9 healthy people were selected as controls (HC). All subjects were scanned with fMRI and DTI. The image data were analysed by local consistency (ReHo), functional connectivity (FC), and spatial statistical method based on fiber tracking (TBSS).

**Results:**

Compared with the migraine group, ReHo in the right dorsolateral middle frontal gyrus, the right inferior frontal gyrus of the insula, and the right inferior frontal gyrus of the trigone in the VM group increased, while the left lingual gyrus, the left posterior cerebellum, and the left fusiform gyrus decreased, and the whole brain FC of the right inferior frontal gyrus in VM group was lower than that in the left middle cingulate gyrus, and lower in the posterior cerebellum than that in the cingulate gyrus. ReHo in the left middle occipital gyrus and left middle temporal gyrus decreased in the VM group compared to the HC group. However, no differences among the three groups were found in DTI.

**Conclusion:**

There may be vestibular multisensory processing abnormalities in patients with VM, while the regulation of cerebellum may be an important role. fMRI detection may open a new sight for VM diagnosis.

## Graphical abstract

Vestibular migraine (VM) exhibits abnormal multisensory processing (increased frontal/insular activation, decreased visual cortex activity) and cerebellar dysregulation, detectable via fMRI. Structural white matter integrity remains unchanged, suggesting functional alterations precede or dominate VM pathology.

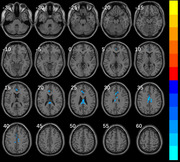

## 1 Introduction

Although the diagnostic criteria only exist in the appendix of ICHD 3 [Headache Classification Committee of the International Headache Society (IHS) 2018], vestibular migraine (VM) has been studied by more and more people since it has had a unified name, especially when it was realized that migraine associated with vertigo/dizziness could have a more reasonable and independent explanation (Von Brevern et al. 2004). As one of the most common vestibular diseases (Lempert et al. 2022), the prevalence of VM is approximately 2.64%, 3.13%, and 1.07% among White, Black, and Asian populations, based on a meta‐analysis conducted in 2020 (Paz‐Tamayo et al. 2020). In addition, it was found by a multi‐center registration that VM accounts for approximately 10.3% of neurological outpatient patients (Cho et al. 2016).VM has had a certain impact on the daily work and life of patients. Ak et al. (2022) found that the quality‐of‐life score of VM patients was lower than that of the healthy group, and anxiety and depression related scores were higher. They also found that the frequency or severity of dizziness or headache attacks was related to the above scores. In addition, it was discovered by Balci et al. (2018) that cognitive function was impaired in VM patients; furthermore, Rizk et al. (Donaldson et al. 2021) found that the degree of vertigo can affect the degree of cognitive impairment. Thus, it is suggested that the power of VM for the impact on the patient cannot be underestimated. However, the mechanism of VM still remains unclear, while the treatment is mainly based on the management of migraine, let alone the challenges of VM diagnosis, our understanding of VM still has a long way to go.

Since 2012, the Bárány society first proposed the diagnostic criteria of VM, and classified it into pVM and dVM, which are shown in Figure S1 (Lempert et al. 2012), while it was dVM that was only included in the appendix of ICHD 3. Seen in Figure S1, it is obvious that the clinical characteristics of VM are recurrent vestibular symptoms dominated by various vertigo, with or without migraine. However, the diagnostic criteria completely rely on clinical manifestations, even the latest version published by the Bárány society in 2022 (Von Brevern et al. 2004) was the same as the last one. In the past ten years, we have seen the dilemma of diagnosis, one of which is the lack of relatively objective laboratory indicators. Although researchers have tried to find such evidence in various aspects, the results have shown that the existing means, such as vestibular function tests (Zaleski et al. 2015) like caloric tests, and different kind of cytokine levels in peripheral blood (Karaaslan et al. 2020) etc., were not sufficient for its diagnostic ability. In spite of actual differences found between VM patients and healthy controls (HC), further researches are still required, considering that the reasons such as insufficient sample size, the influence of confounding factors may exceed the influence of disease (Perez‐Carpena and Lopez‐Escamez 2021); however, these methods still have the value in differential diagnosis (Rizk et al. 2020), and also broaden the thoughts of understanding and studying the mechanism of VM.

Imaging examinations are certainly one of the research hotspots, especially with the accelerated development of fMRI, DTI, and other related imaging technologies in recent years. As a means of noninvasive analysis of brain function, fMRI has been widely studied in the pathophysiological mechanisms of various nervous diseases, including VM. Compared with task state, resting state fMRI is easier to implement, with great repeatability, and has abundant content to analyze. It has obvious advantages for studying the spontaneous activity of the brain and the functional connection between brain regions. ReHo value better reflects the local synchronization of spontaneous neural activity. Functional connectivity (FC) can reflect the functional correlation between different brain regions in space; the above two analysis methods are most widely used in the study of VM. As early as 2014, Russo et al. (2014) performed task‐based functional magnetic resonance imaging (fMRI) on 12 patients with VM during cold water ear irrigation, the results showed that compared with patients with migraine without aura and HC, the activation of thalamus in VM patients was significantly increased, while the activation degree was positively correlated with the frequency of migraine attacks in patients. Yu et al. (2017) also obtained similar findings. As for a further result, a resting state functional MRI study carried out by Chen et al. (2022), it was found that compared with the healthy control group, the FC between the left thalamus and the left anterior cingulate cortex (ACC), bilateral insula, and right auxiliary motor cortex in VM patients decreased, together with the FC between the right thalamus and the left insula and ACC, but the FC between the left thalamus and the right precuneus and middle frontal gyrus, and between the right thalamus and the superior parietal lobule increased, which suggested that VM patients have reduced thalamic pain and thalamus‐vestibular pathway, while showing enhanced thalamic visual pathway. The findings of fMRI in VM patients are of certain value in understanding the disease, studying the mechanism, and exploring relatively objective diagnostic indicators. DTI can reflect the degree of damage to white matter fiber bundles by detecting the changes in the diffusion parameters of water molecules in brain tissue. Among them, tracer‐based spatial statistics (TBSS) is a voxel‐based method for analyzing white matter fibers, which is sensitive to the changes in white matter, but there is still a lack of research on the changes in VM fiber bundles.

In this study, we used the DTI based spatial statistical method of tractography (TBSS), and ReHo and FC of resting state fMRI to analyze the characteristics of brain structure and function changes in patients with VM.

## 2 Materials and Methods

### 2.1 Participants

During February 2022 to January 2023, there were finally 13 patients with VM and 12 with migraine from the neurology outpatient clinic of the First Affiliated Hospital of Harbin Medical University, were included in this study. Nine healthy individuals with gender match were selected as the healthy control group. All the patients were diagnosed by the same experienced neurologist according to the diagnostic criteria of VM from the Bárány society and migraine from ICHD 3. Participants were aged between 18 and 65 years. The specific exclusion criteria are as follows: having contraindications for MRI examination, people with other episodic vertigo disorders (e.g. Meniere's disease, benign positional vertigo, transient ischemic attacks in the posterior circulation, vestibular paroxysmal syndrome, etc.), people who have other disease history (e.g. psychosocial related diseases, cerebrovascular diseases, tumors, hyperthyroidism, diabetes and other endocrine system related diseases, hepatitis, AIDS and other infection related diseases, cardiovascular diseases, hematologic diseases, liver and kidney dysfunction, etc.), under a state of stress, women who are at pregnancy and lactation, alcohol and drugs abusers, and people who refuse to collaborate. The general information of each subject was collected by the same neurological resident, such as age, gender, education level, and etc. As for participants with VM or migraine, history, like the course, frequency, duration, and severity of vertigo and headache, were recorded. Written informed consent was obtained from all subjects, and the study was approved by the Ethics Committee of the First Affiliated Hospital of Harbin Medical University.

### 2.2 Data Collection

All the MRI data were acquired on the same scanner (3.0 T, Philips Achieva) and performed by an experienced examiner. During the examination, participants were informed to stay still while there was a special tool to limit head movement, keep their eyes closed, avoid emotional lability, and mental activity. Considering its high temporal resolution, the resting state BOLD fMRI data in this study was obtained through Echo Planar Imaging (EPI), in which the specific scanning parameters were as follows: fMRI scanning time was 12 min, repetition time was 3s, echo time was 35 ms, scanning resolution was 64 × 64, layer thickness was 3.5 mm, flip angle was 90°, scanning field of view was 224 mm × 224 mm, voxel was 3.5 mm × 3.5 mm × 3.5 mm. We used a three‐dimensional gradient echo T1WI sequence to obtain sagittal images of the subjects for registration and standardization of resting state functional magnetic resonance images, the parameters were as follows: repetition time was 9.9 ms, echo time was 4.6 ms, layer thickness was 2 mm, flipping angle was 90°, scanning field was 256 mm × 256 mm, voxel was 1.0 mm × 1.0 mm × 2.0 mm. The DTI data parameters are as follows: repetition time was 3931 ms, echo time was 93 ms; Flip angle was 90°, field of view was 224 mm × 224 mm × 120 mm, voxel was 2.0 mm × 2.0 mm × 2.0 mm, matrix was 112 × 110, layer thickness was 2 mm. The diffusion gradient scheme was configured using the scanner's default “medium resolution” protocol (15 directions). The total scanning time is 25 min and 31 s.

### 2.3 Image Preprocessing

#### 2.3.1 Images of fMRI

In order to achieve subsequent analysis, it needs to perform a series of steps to improve the quality of data, which is called image preprocessing, and DPABI 6.0 has been used in this study. It includes the following steps: (1) Format conversion: Convert the structure image and function image from the initial format to NIFTI format data; (2) Remove interference: Remove the data of the first five time points in the function image; (3) Slice time correction: Also called Slice Timing; (4) Motion correction: To correct head movement during scanning; (5) Image registration: Manual spatial registration of structure image and function image of each case; (6) Image segmentation: Peel off the scalp and segment the image into gray matter, white matter and cerebrospinal fluid; (7) Space normalization: According to the segmentation result of the structure image, the function image is converted to the MNI standard space; (8) Filtering: Around 27 unrelated covariates (including 24 head movement parameters, gray matter, white matter, and the average value of the whole brain) were removed from the functional data; (9) Smoothing: Use a smoothing kernel of [4,4,4] to blur data in space to reduce the impact of spatial changes; (10) Artifact detection and correction: Identify and correct artifacts in data caused by head movement, physiological noise or scanner failure; (11) Obtain mask: Create a mask that excludes non‐brain regions from analysis; (12) Quality control: Conduct visual inspection on data to ensure the success of pretreatment steps and obtain high quality of data.

#### 2.3.2 Images of DTI

This process was mainly completed by using the FSL toolkit. The steps are as follows: (1) Format conversion: Convert the original DTI data to the standard NIFTI format; (2) Extracting b0 image; (3) Brain extraction: Use brain extraction tools to remove redundant tissues from images; (4) Eddy current correction: Correct the image distortion caused by scanner magnetic field; (5) Diffusion tensor estimation: use the definition function based on the diffusion tensor model to estimate the diffusion tensor of each voxel in the brain; (6) Calculate diffusion parameters: calculate the eigenvalues of each voxel, including fractional anisotropy (FA), mean diffusivity (MD), and other parameters. (7) Fiber tracking: The white matter tract is reconstructed using diffusion data, and nerve fiber tracking is performed.

### 2.4 Data Analysis of Images

After fMRI data preprocessing and index calculation, the images of relevant indicators of the three groups of data were obtained. The VM group and migraine group data were analyzed by two sample *t*‐tests. The details are as follows:

Based on the null hypothesis (we assume that the average value of VM and migraine population on a certain index is equal) and the current VM sample and migraine sample, the *t* value was calculated, and the calculation formula is as follows:

$$t=\frac{\left(\bar{x}-\bar{y}\right)}{\sqrt{\frac{s_{1}^{2}}{n_{1}}+\frac{s_{2}^{2}}{n_{2}}}}$$

where $\bar{x},\bar{y}$ represents the mean value of each voxel in VM and migraine on a certain index; $s_{1},\;s_{2}$ are the standard deviation of each voxel in VM and migraine on a certain index, while $n_{1},n_{2}$ are the sample size of VM and migraine. In this study, $n_{1}=13,\;n_{2}=12$.

The degree of freedom was calculated through the following formula:

$$df=\frac{{\left(\frac{s_{1}^{2}}{n_{1}}+\frac{s_{2}^{2}}{n_{2}}\right)}^{2}}{\frac{1}{n_{1}-1}{\left(\frac{s_{1}^{2}}{n_{1}}\right)}^{2}+\frac{1}{n_{2}-1}{\left(\frac{s_{2}^{2}}{n_{2}}\right)}^{2}}$$

The *p* value was obtained by querying chi square distribution table according to the corresponding degree of freedom; it was statistically significant, while *p *< 0.05.

Statistical analysis was based on the standard of *p *< 0.05 and voxel cluster size >15 (405 mm^3^), to obtain the regional location where the relevant indicators of the vestibular migraine group and migraine group decrease or increase in the resting state. There were two important indicators, including local consistency ReHo and functional connection FC, in which the following statistics and analysis have been carried out.

#### 2.4.1 ReHo Analysis

The Kendall coefficient of concordance (KCC) is a measurement method of correlation, and ReHo was mainly measured by the KCC. The following are the calculation formulas:

(1) $$KCC=\frac{\sum {\left(R_{i}\right)}^{2}-n{\left(\bar{R}\right)}^{2}}{\frac{1}{12}K^{2}\left(n^{3}-n\right)}$$

KCC represents the Kendall coefficient of concordance of voxels, with the value of [0, (IHS) 2018)]. *K* is the adjacency mode of voxels, and the values are 7, 19, and 27 in general. *n* represents the number of data points in the time series. In this study, most of the collected data are 240 time points, with the first five time points removed after preprocessing; therefore, *n* = 235. $R_{i}$ is the sum of the series of the $No._{i}$ time point. $\bar{R}$ in Formula (1) is the mean value of $R_{i}$, which can be calculated by the Formula (2):

(2) $$\bar{R}=\frac{K\left(n+1\right)}{2}$$

According to Formulas (1) and (2), the ReHo of each voxel in the whole brain can be calculated. The larger the KCC value is, the higher the time consistency of local brain areas is, that is, the higher the synchronization of neural activities is, and vice versa. ReHo values were first calculated from unsmoothed BOLD time series using DPABI in MATLAB. The resulting ReHo maps were then spatially smoothed to generate final images for statistical comparisons. The ReHo maps of the VM group and migraine group were compared and analyzed by two sample *t*‐test and corrected by threshold‐free cluster enhancement (TFCE). After correction, the brain regions with statistically significant differences were obtained according to the standard of voxel cluster level (*p *< 0.05) and voxel cluster size >15 (405 mm^3^).

#### 2.4.2 FC Analysis

Based on the results of ReHo, brain regions with obvious differences were selected as seed points for FC analysis, and the correlation between seed points and whole brain voxels was calculated. Two‐sample *t*‐test was conducted for the VM group and migraine group after being corrected by TFCE, with the standard of voxel cluster level *p *< 0.05 and voxel cluster size >15 (405 mm^3^) to obtain the brain regions with different FC.

### 2.5 Statistical Analysis

In all analyses, the number of permutation test is 5000. At the cluster level, we used TFCE for multiple comparison correction. The general data of the three groups were statistically analyzed by SPSS 25.0. Fisher's precision probability test was used for gender comparison between groups. Age and education years were compared by *t*‐test, using average ± standard deviation $\bar{x}\pm s$. The significance level *α* = 0.05, and *p *< 0.05 meant statistically significant. The VM group and the healthy control group were analyzed in the same way. Furthermore, to assess the relationship between attack frequency and ReHo, Spearman's rank correlation analysis was performed using GraphPad Prism. Attack frequency was categorized as follows: 1 = monthly episodes (highest frequency), 2 = several‐monthly episodes, and 3 = yearly episodes (lowest frequency). Brain regions with significant ReHo differences between the VM group and the healthy control group were selected from the above *t*‐test for correlation analysis.

## 3 Results

### 3.1 Demographic and Clinical Characteristics

As shown in Table 1, there were no significant differences (*p *< 0.05) between the three groups in demographic characteristics like age, years of education, etc. All patients were in the interictal period.

**TABLE 1:** Comparison of demographic and clinical characteristics among the three groups.

	VM group	Migraine group	Healthy control group	*p value	*^#^p* value	*^p* value
Age	37.92 ± 8.72	38.54 ± 9.91	39.44 ± 9.28	0.868	0.700	0.831
Gender(males/females)	(12/1)	(11/1)	(7/2)	1.000	0.544	1.000
Yeas of education (year)	11.54 ± 2.44	12.38 ± 2.26	11.78 ± 2.635	0.367	0.829	0.569
Duration of disease(year)	5.08 ± 2.60	5.77 ± 3.00				
Headache frequency (times per year)	4.92 ± 2.06	8.92 ± 2.60				
VAS scores	4.38 ± 1.61	6.62 ± 1.56				
DHI scores	44.00 ± 4.46					

*Note*: (1) Age follows normal distribution, using mean ± standard deviation; (2) * p value indicates the comparison between VM group and migraine group; *^#^ p* value indicates the comparison between VM group and healthy control group; *^ p* value indicates the comparison between the migraine group and the healthy control group.

### Results of ReHo

**FIGURE 1:**
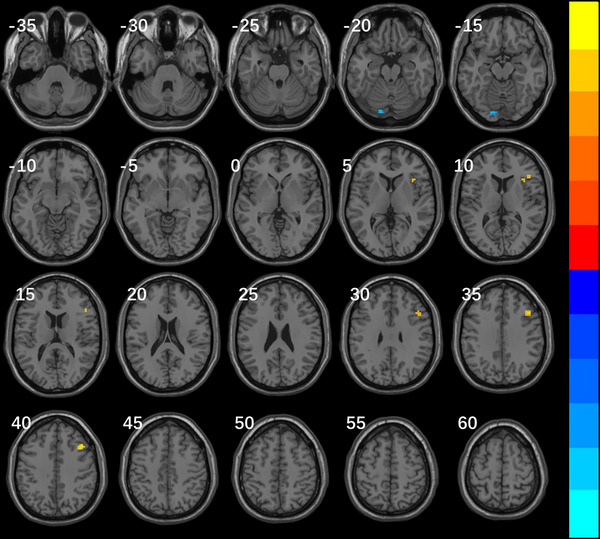
Brain regions with global ReHo differences between VM and migraine groups Note: Significantly different regions in ReHo between patients with VM and migraine groups: TFCE corrected voxel cluster level *p *< 0.05 and voxel cluster size>15. Compared with the migraine group, the ReHo of VM group was increased in the right dorsolateral middle frontal gyrus, right inferior frontal gyrus of insular cortex and right inferior frontal gyrus of triangle (warm color), and decreased in the left lingual gyrus, left posterior cerebellar and left fusiform gyrus (cool color). The color bars represent T‐values, corresponding to the magnitude of cluster peaks. Warm colors (e.g., red‐yellow) denote positive values, while cool colors (e.g., blue‐cyan) indicate negative values. Lighter hues reflect larger absolute values.

**FIGURE 2:**
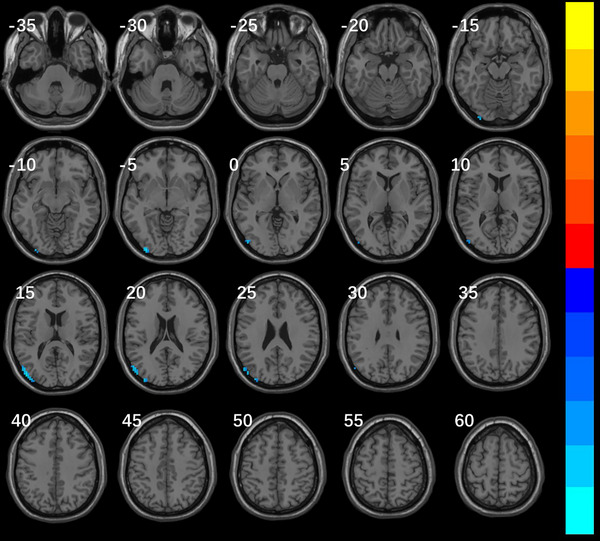
Brain regions of global ReHo differences between VM group and HC group Note: Significantly different regions in ReHo between patients with VM and HC groups: TFCE corrected voxel cluster level *p* < 0.05 and voxel cluster size>15. The VM group had reduced ReHo (cold color) in the left medial occipital gyrus and temporal gyrus compared with the HC group. The color bars represent T‐values, corresponding to the magnitude of cluster peaks. Warm colors (e.g., red‐yellow) denote positive values, while cool colors (e.g., blue‐cyan) indicate negative values. Lighter hues reflect larger absolute values.

Compared with the migraine group, ReHo in the right dorsolateral middle frontal gyrus, the right inferior frontal gyrus of the insula, and the right inferior frontal gyrus of the trigone in the VM group increased, while the left lingual gyrus, the left posterior cerebellum, and the left fusiform gyrus decreased (Table 2, Figure 1). ReHo decreased in the left middle occipital gyrus and left middle temporal gyrus in the VM group compared with the healthy control group (Table 3, Figure 2).

**TABLE 2:** Brain regions of global ReHo differences between VM and migraine groups.

Brain regions	Voxels	Peak MNI coordinates (X Y Z in mm)	*t* value
Cluster1	34	39 21 39	5.46853
Right dorsolateral middle frontal gyrus	25		
Right inferior frontal gyrus of the insula	5		
Right inferior frontal gyrus of the trigone	4		
Cluster2	24	−12 −87 −18	−6.9666
Left lingual gyrus	11		
Left posterior cerebellum	10		
Left fusiform gyrus	3		

*Note*: The brain regions in the results were all located based on the AAL3 standard template; MNI means Montreal Neurological Institute; Peak MNI is the coordinate of the location of the peak of the voxel cluster; *t* value equals to voxel cluster peak, the positive value indicates that the activity of VM group is higher than that of migraine group, and a negative value indicates that the activity is lower.

**TABLE 3:** Brain regions of global ReHo difference between VM group and HC group.

Brain regions	Voxels	Peak MNI coordinates (X Y Z in mm)	*t* value
Cluster 1	85	−48 −84 15	−6.2181
Left middle occipital gyrus	24		
Left middle temporal gyrus	6		
Cluster 2	21	−33 −96 −6	−5.7106
Left middle occipital gyrus	9		

*Note*: The brain regions in the results were all located based on the AAL3 standard template; MNI means Montreal Neurological Institute; Peak MNI is the coordinate of the location of the peak of the voxel cluster; *t* value equals to voxel cluster peak, the negative value represents the reduction of ReHo in VM group and healthy control group.

### 3.2 Results of FC

**FIGURE 3:**
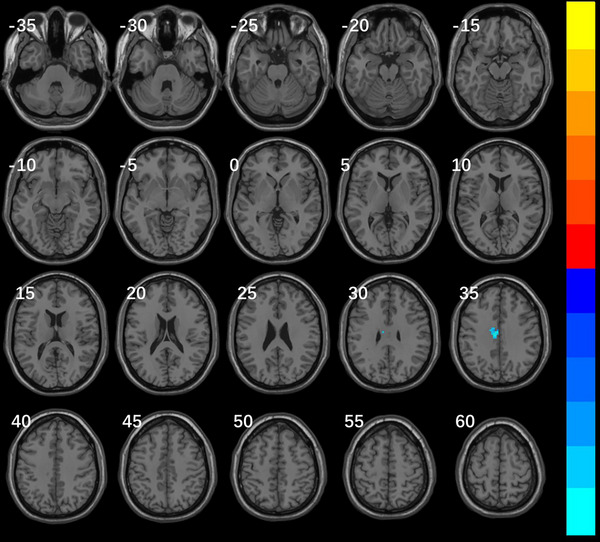
Whole brain FC map of differences between VM with ROI based on right triangular inferior frontal gyrus and migraine group Note: Significantly different of brain FC map between patients with VM and migraine groups: TFCE corrected voxel cluster level *p* < 0.05 and voxel cluster size>15. The global FC of the VM group and the right inferior frontal gyrus of the trigone was lower in the left middle cingulate gyrus than in the migraine group (cold color). The color bars represent T‐values, corresponding to the magnitude of cluster peaks. Warm colors (e.g., red‐yellow) denote positive values, while cool colors (e.g., blue‐cyan) indicate negative values. Lighter hues reflect larger absolute values.

**FIGURE 4:**
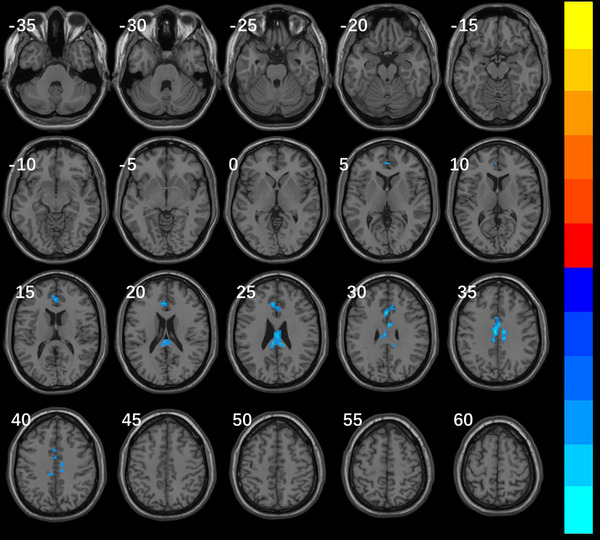
Whole brain FC map of differences between VM with ROI in posterior cerebellum and migraine group Note: Significantly different of brain FC map between patients with VM and migraine groups: TFCE corrected voxel cluster level *p* < 0.05 and voxel cluster size>15. The whole brain FC in the posterior cerebellum of VM group was lower than that of migraine group in the left middle cingulate gyrus, right middle cingulate gyrus, upper part of left anterior cingulate gyrus, anterior part of left anterior cingulate gyrus, upper part of right anterior cingulate gyrus, left posterior cingulate gyrus, right posterior cingulate gyrus (cold color). The color bars represent T‐values, corresponding to the magnitude of cluster peaks. Warm colors (e.g., red‐yellow) denote positive values, while cool colors (e.g., blue‐cyan) indicate negative values. Lighter hues reflect larger absolute values.

Select the brain region with an obvious difference in the above three indicators as the region of interest, and compare the characteristics of the FC with the whole brain. It was shown that there were differences in the FC of the whole brain in the right inferior frontal gyrus and posterior cerebellum between the VM group and migraine group. (Table 4, Figure 3, Figure 4)

**TABLE 4:** Whole brain FC results of VM group and migraine group based on ROI of right inferior frontal gyrus of the trigone and posterior cerebellum.

Brain regions	Voxels	Peak MNI coordinates (X Y Z in mm)	*t value*
Right inferior frontal gyrus of the trigone			
Cluster 1	39	−6 −18 36	−5.7564
Left middle cingulate gyrus	25		
Posterior cerebellum			
Cluster 1	301	−3 15 33	−5.9907
Left middle cingulate gyrus	75		
Right middle cingulate gyrus	52		
Upper part of left anterior cingulate gyrus	46		
Anterior part of left anterior cingulate gyrus	20		
Upper part of right anterior cingulate gyrus	8		
Left posterior cingulate gyrus	7		
Right posterior cingulate gyrus	4		

*Note*: The brain regions in the results were all located based on the AAL3 standard template; MNI means Montreal Neurological Institute; Peak MNI is the coordinate of the location of the peak of the voxel cluster; *t* value equals to voxel cluster peak, the positive value indicates that the activity of VM group is higher than that of migraine group, and a negative value indicates lower.

### 3.3 Results of DTI

There were no significant differences in the TBSS analysis of DTI between VM group, migraine group, and HC group.

### 3.4 ReHo Correlates With Attack Frequency in VM

**FIGURE 5:**
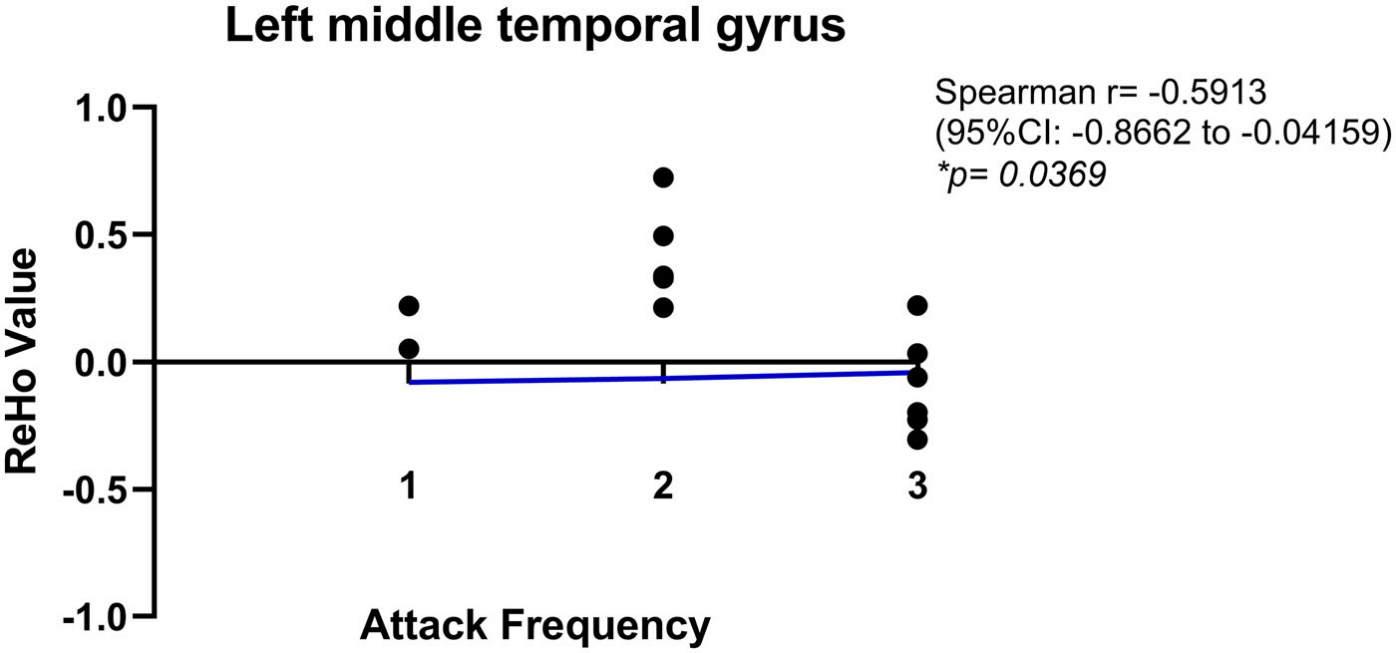
Analysis of correlation between regional homogeneity in left middle temporal gyrus and attack frequency in vestibular migraine. Scatterplot of ReHo in the left middle temporal gyrus versus clinical attack frequency stratification (1: annual episodes; 2: monthly episodes; 3: weekly episodes). The blue regression line demonstrates a significant inverse correlation (Spearman's *ρ* = −0.5913, *p* = 0.0369), with higher attack frequencies corresponding to reduced ReHo values.

**FIGURE 6:**
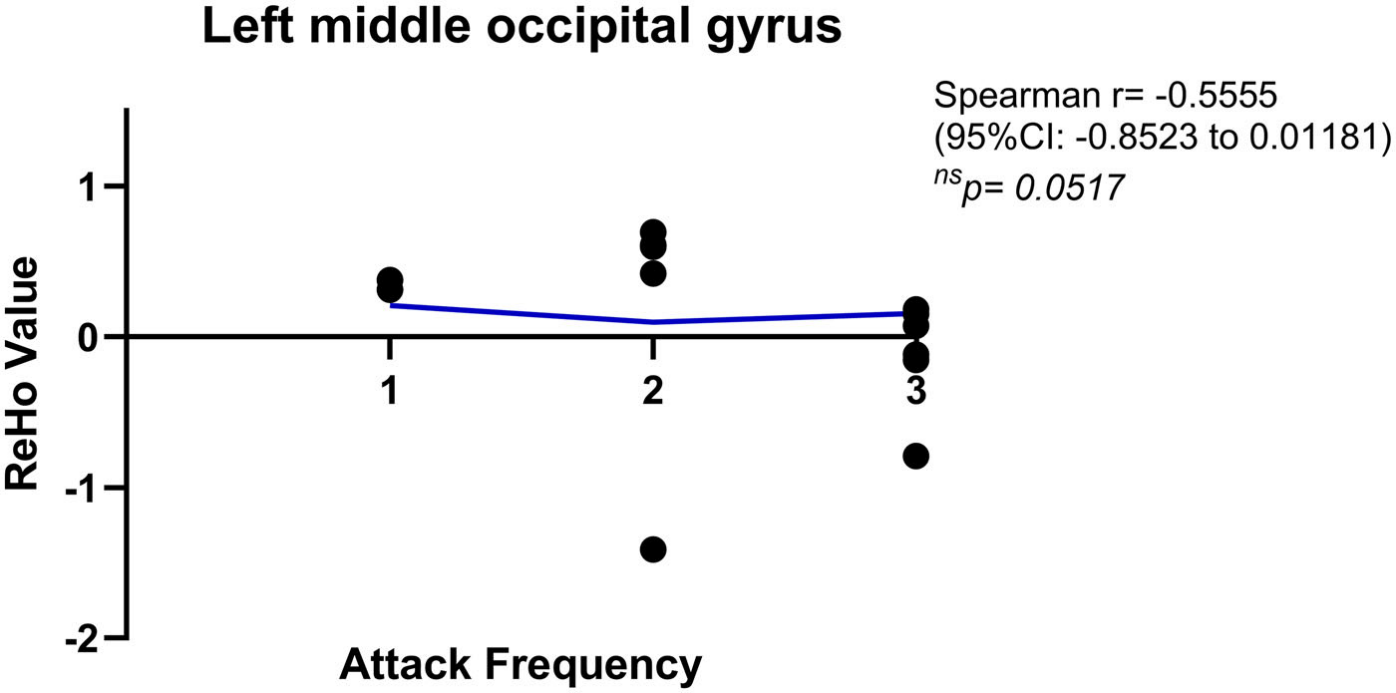
Analysis of correlation between regional homogeneity in left middle occipital gyrus and attack frequency in vestibular migraine. Scatterplot of ReHo in the left middle occipital gyrus versus clinical attack frequency stratification (1: annual episodes; 2: monthly episodes; 3: weekly episodes). The blue regression line demonstrates a significant inverse correlation (Spearman's *ρ* = −0.56, *p* = 0.052), with higher attack frequencies corresponding to reduced ReHo values.

ReHo values in the left middle temporal gyrus and left middle occipital gyrus were extracted from the VM group and correlated with attack frequency. A significant negative correlation was observed between ReHo in the left middle temporal gyrus and attack frequency (Spearman's *r* = −0.59, 95% CI: −0.87 to −0.04,  *p* = 0.037; Figure 5). Although no significant association was found in the left middle occipital gyrus (Spearman's *r* = −0.56, 95% CI: −0.85 to −0.01, *p* = 0.052; Figure 6), the effect direction suggested a potential negative trend.

## 4 Discussion

### 4.1 Patients With VM Had Significant Frontal Activation

Compared with migraine group and the HC group, the VM group has been found to have more obvious activation in the frontal lobe, including some regions of the middle frontal gyrus and inferior frontal gyrus. It is reported that PFC in the prefrontal cortex is the most critical brain region related to structural changes in migraine patients (Jia and Yu 2017). PFC participates in the attention network in which its functions involve cognition, thinking, and emotion processing, while the middle frontal gyrus is a significant part of it. Long‐term and recurrent attacks in VM patients may lead to cognitive decline. Previous studies have found that among the patients with the worst performance in the visuospatial memory test during neuropsychological evaluation, the activation of the frontal lobe region was more obvious, which indicated that these patients had more obvious adaptive responses (Messina et al. 2021). This study has shown that VM patients had disturbances in perception, cognition, and emotional processing.

### 4.2 Patients With VM Had Significant Right Brain Activation

The activation of the cerebral cortex in the vestibular stimulation experiment showed an obvious right hemisphere advantage. This study showed that the brain regions activated in the VM group were mostly located in the right brain compared with migraine group and the HC group, which is consistent with some studies that believed the vestibular cortical response network had right hemisphere advantage (Hashimoto et al. 2013; Raiser et al. 2020). The middle temporal gyrus, which belongs to the lateral vestibular cortex of the temporal lobe, is a very sensitive area to dizziness. Studies showed that the middle temporal gyrus plays a key role in interconnecting with other multisensory cortical regions (Helmchen et al. 2014; Schwedt et al. 2013). This study revealed that the VM group exhibited decreased ReHo in the left middle temporal gyrus compared to the healthy control group. Furthermore, correlation analysis demonstrated a significant negative association between ReHo in the left middle temporal gyrus and higher attack frequency within the VM group, suggesting potential impairments in vestibular multisensory information processing in these patients, and disease severity may be associated with functional alterations in vestibular multisensory information processing.

### 4.3 Visual Cortex Activity Was Attenuated in Patients With VM

Previous studies have shown that the activation modes of vestibular, visual, auditory, and other sensory systems interact. Especially when the vestibule is stimulated, the visual cortex is generally inhibited. In this study, the activation of the left lingual gyrus, fusiform gyrus, and other occipital cortex decreased in the VM group rather than in migraine group. Considering that those areas are mostly related to visual processing, which indicates it might be the result of multisensory interaction and mutual inhibition of the visual processing system and vestibular processing system.

### 4.4 Differences in Cerebellar Activity and FC in Patients With VM

As a center of pain regulation, emotional, and sensorimotor processing, the cerebellum is involved in the functional integration of multiple systems. There are more and more structural and functional neuroimaging studies that have shown that the cerebellum is involved in the occurrence of migraine, especially Crus I, II, and lobule VI of the vermis (Kros et al. 2018). Mehnert and May (2019) discovered that the periaqueductal gray matter and the posterior cerebellum (Crus I, II area) are co‐activated during the pain stimulation to the trigeminal in migraine patients. It was shown that the motor and cognitive functions of the cerebellum come from the integration of vestibular signals to the inner ear, and Purkinje cells in the cerebellar cortex project to the vestibular nucleus in the dorsal region of the pons. Thus, changes in the cerebellar vestibular pathway may lead to vertigo, dizziness, and balance disorders, which could be related to vestibular migraine (Noseda 2022). Shin et al. (2014) found that the functional connection between the right cerebellum and the right putamen is significantly enhanced in patients with VM, while bilateral cerebellar activation occurs during the attacks, which suggests that cerebellar dysfunction may be involved in the pathogenesis of VM. As for Crus I and II area, previous studies have discovered that those regions are closely related to the associative cortex, especially the high‐level cognitive regions like prefrontal and posterior parietal cortex, which are involved in cognitive and emotional functions (Moulton et al. 2010). Although no significant differences in FC were detected between the VM group and HC in our study, this discrepancy may be attributed to the limited sample size and the selection/definition of ROIs. In contrast, Zhe, Zhang, et al. (2021) identified increased FC between the primary somatosensory cortex, inferior parietal lobule, and left parieto‐insular vestibular cortex (superior temporal gyrus) in VM patients compared to HC, based on gray matter volume‐derived ROIs and voxel‐wise analyses. In our study, the VM group showed weaker activity in the left posterior cerebellum compared to the migraine group, which indicates the role of the cerebellum in migraine and VM. Furthermore, it is known that the dominant vestibular hemisphere is on the right side, and the weakened activity of the left cerebellum suggests the intersection of information between the cerebellum and the cerebral hemisphere. Liu et al. found that the ALFF value of the left posterior cerebellum has significantly increased after vestibular rehabilitation treatment in 14 patients with VM, which also verified this point (Liu et al. 2020).

### 4.5 Changes of Activity and FC in the Cingulate in Patients With VM

The cingulate cortex is the center of cognition and emotion control and plays an important role in pain regulation. The anterior and posterior cingulate gyri are parts of the default mode network. The anterior cingulate gyrus is closely connected with regions related to emotion processing, motivation, and autonomic functions, to play corresponding functions. The middle cingulate gyrus is considered to be part of the salience network, mainly connecting with regions that are related to action control and decision‐making, such as dorsolateral PFC, sensorimotor cortex, pontine nucleus, and periaqueductal gray matter (Van Heukelum et al. 2020). This research showed that the VM group had higher activities in the anterior cingulate gyrus and middle cingulate gyrus than the HC group, and the FC in the posterior cerebellum of the VM group was lower than that of the migraine group in the cingulate gyrus. Similarly, Li et al. (2022) employed a functionally refined but anatomically coarse parcellation of brain networks in their fMRI study of VM. For instance, the posterior default mode network (pDMN) primarily included the posterior cingulate cortex, precuneus, and bilateral lateral parietal cortices. Under this framework, they observed reduced FC within the sensorimotor network, specifically between the bilateral medial cingulate gyrus and paracentral lobule in VM patients. It may be related to the pain processing dysfunction, the interruption of perception and regulation, and the compensatory increase of cingulate function caused by repeated pain attacks.

### 4.6 No Significant Differences Have Been Found in DTI

The results of DTI in this study seemed to show a disappointing outcome; however, while there is no related research for VM, we speculate that some factors should be reasonably considered. It is possible that the changes in patients are not enough to be recognized, and as image differences in the DTI. Also, the problem of insufficient sample size needs to be considered. After all, there were different results in previous DTI studies for migraine patients. Yang et al. (2022) used resting state fMRI to study the FC of thalamic subregions in patients with episodic migraine and applied DTI to analyze the corresponding structural changes, which found that there was no significant difference in DTI parameters between the VM group and the healthy control group. It is also discovered by Lars et al. (Neeb et al. 2015) that there was no statistically significant difference in DTI parameters between patients with chronic migraine or episodic migraine and HC. However, Álvaro et al. (Planchuelo‐Gómez et al. 2020) found that the onset time of chronic migraine was significantly positively correlated with the average anisotropy fraction of bilateral external capsules while it was significantly negatively correlated with the average radial diffusivity of bilateral external capsules, which means that compared with episodic migraine, axonal integrity damage may occur in the first few months of chronic migraine. Previous studies have indicated another issue worthy of attention, that is, the disease state of patients, long‐term and chronic attacks may accumulate over time and gradually produce irreversible structural damage. Certainly, this needs to be verified by further studies.

### 4.7 Limitations

Our study has several limitations that warrant consideration: (1) The relatively small sample size and lack of strict control over confounding factors (e.g., medication status) may compromise the reproducibility of the results. Future studies with larger cohorts are required to validate these findings; (2) the heterogeneity of VM subtypes was not addressed. Enlarging the sample size in subsequent research will enable exploration of fine‐grained functional alterations across distinct clinical phenotypes; (3) The absence of a control group comprising migraine patients without vestibular symptoms limits pathophysiological specificity. This will be rectified in our ongoing longitudinal study.

## 5 Conclusion

(1) The increased activity of the right frontotemporal and insular vestibular cortex and the decreased activity of the occipital visual cortex in patients with VM further suggest the pathogenesis of vestibular multisensory processing abnormalities in patients with VM; (2) the regulation of cerebellum may be one of the mechanisms of VM; (3) fMRI detection may open up a new research direction for VM diagnosis; (4) The changes of brain fiber bundles in patients with VM may not be related to their functional changes, or lag behind their functional changes.

## Author Contributions

**Ni Liu**: conceptualization, investigation, writing – original draft, methodology, validation, visualization, writing – review and editing, software, project administration, formal analysis, data curation, supervision. **Qijun Yu**: conceptualization, investigation, writing – original draft, methodology, validation, visualization, writing – review and editing, software, project administration, formal analysis, data curation, supervision. **Shaowei Gan**: investigation, methodology, validation, visualization, writing – review and editing, software, data curation, formal analysis, project administration, supervision, resources. **Yonghui Pan**: conceptualization, funding acquisition, methodology, writing – review and editing, formal analysis, project administration, supervision, resources. **Zhaowen Qiu**: writing – review and editing, methodology, software, formal analysis, project administration, data curation, supervision.

## Peer Review

The peer review history for this article is available at https://publons.com/publon/10.1002/brb3.70569

## Supporting information



Figure S1. Diagnostic criteria of Vestibular migraine from Bárány Society

## Data Availability

The data that support the findings of this study are available on request from the corresponding author. The data are not publicly available due to privacy or ethical restrictions.
