# Supplementary figures and images for: Functional and Structural Differences of Brain in Patients With Vestibular Migraine: A Resting‐State Functional MRI and DTI Study
Source: Brain Behav. 2025 Jun 12;15(6):e70569. doi: 10.1002/brb3.70569 (PMC12159766; doi:10.1002/brb3.70569)

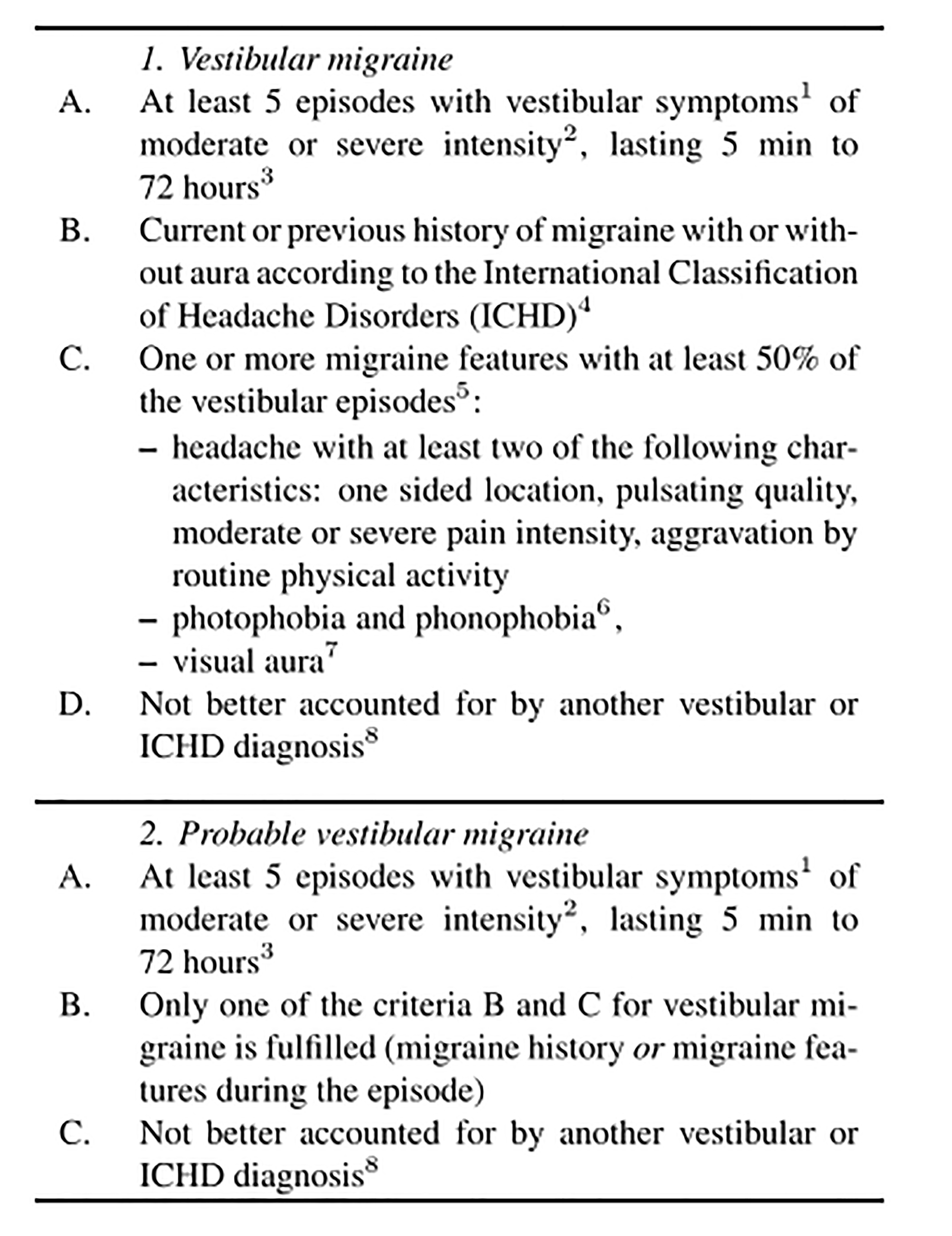

Supplement: Supplementary file 1 — Figure S1. Diagnostic criteria of Vestibular migraine from Bárány Society [file BRB3-15-e70569-s001.jpg]
